# Commentary on Unnecessary reliance on multilevel modelling to analyse nested data in neuroscience: When a traditional summary-statistics approach suffices

**DOI:** 10.1016/j.crneur.2022.100041

**Published:** 2022-05-20

**Authors:** Paul Alexander Bloom, Monica Kim Ngan Thieu, Niall Bolger

**Affiliations:** Department of Psychology, Columbia University, New York, NY, USA

McNabb and Murayama (2021) present a set of simulations demonstrating that under certain circumstances, simpler models based on summary statistics perform equivalently to multilevel models (MLM). We commend the clarification that the summary statistic approach does not automatically result in a loss in statistical power and agree that the summary-statistics approach can be useful in some cases in neuroimaging (Mumford and Nichols, 2009).

However, the authors' conclusion that the summary statistics approach can do “an equally good or even better job in some situations” overstates the drawbacks of MLM and neglects its key advantages. Most importantly, the singular fit errors cited by the authors as common with MLM arise only in frequentist methods that use maximum likelihood estimation (e.g. lmer; Bates and Bolker, 2011). With Bayesian MLM methods that use Markov-Chain Monte Carlo computations, such singular fit or convergence errors largely do not occur. Bayesian multilevel regression models can be readily fit using syntax nearly identical to lmer using the brms (Bürkner, 2017) and rstanarm (Gabry et al., 2019) packages in R, or with the PyMC python library (Salvatier et al., 2016). Thus, Bayesian MLM methods can alleviate the singular fit errors and associated issues with power and Type I error rates that can occur with equivalent maximum-likelihood MLM methods.

Further, although in certain circumstances the summary-statistics approach returns equivalent results to MLM fixed effects (Murayama et al., 2022), the summary-statistics approach can be problematic in cases of unequal cluster sizes or unequal cluster variances (Gelman and Hill, 2006). This approach tends to overweight summary statistics from smaller clusters in estimating fixed effects (Gelman, 2006). In contrast, the partial pooling of variances across clusters within the MLM framework can improve estimation of uncertainty and model predictive performance (van Boekel, 2021).

Finally, though the approaches can be equivalent in detecting fixed or average effects, the summary-statistics approach is unable to estimate the extent of between-cluster variation in those effects. Unless within-cluster variance is explicitly modeled (for example, by using a “sufficient” summary-statistic approach; see Dowding and Haufe, 2018), between-cluster variation in effects will be overestimated. By contrast, MLM approaches give researchers a principled set of tools for understanding between-person and other sources of effect heterogeneity (Bolger et al., 2019; Chen et al., 2021; Koo and Li, 2016). When effects vary widely across participants, for example, methods that only focus on the average participant can be very misleading (DiGiovanni et al., 2021; Haaf and Rouder, 2019). Thus, summary-statistics approaches, by discarding information on random effects, can impede researchers’ understanding of the data-generating process and the development of adequate theories (Baayen et al., 2008). Thus, we encourage the broad use of MLMs, particularly within a Bayesian framework, when possible.

## Declaration of competing interest

The authors declare that they have no known competing financial interests or personal relationships that could have appeared to influence the work reported in this paper.
